# Public Disclosure of Results From Artificial Intelligence/Machine Learning Research in Health Care: Comprehensive Analysis of ClinicalTrials.gov, PubMed, and Scopus Data (2010-2023)

**DOI:** 10.2196/60148

**Published:** 2025-03-21

**Authors:** Shoko Maru, Ryohei Kuwatsuru, Michael D Matthias, Ross J Simpson Jr

**Affiliations:** 1 Real‑World Evidence and Data Assessment (READS) Graduate School of Medicine Juntendo University Tokyo Japan; 2 Department of Radiology School of Medicine Juntendo University Tokyo Japan; 3 Matthias IT Pty Ltd Brisbane Australia; 4 Division of Cardiology School of Medicine University of North Carolina Chapel Hill Chapel Hill, NC United States

**Keywords:** machine learning, ML, artificial intelligence, AI, algorithm, model, analytics, deep learning, health care, health disparities, disparity, social disparity, social inequality, social inequity, data-source disparities, ClinicalTrials.gov, clinical trial, database, PubMed, Scopus, public disclosure of results, public disclosure, dissemination

## Abstract

**Background:**

Despite the rapid growth of research in artificial intelligence/machine learning (AI/ML), little is known about how often study results are disclosed years after study completion.

**Objective:**

We aimed to estimate the proportion of AI/ML research that reported results through ClinicalTrials.gov or peer-reviewed publications indexed in PubMed or Scopus.

**Methods:**

Using data from the Clinical Trials Transformation Initiative Aggregate Analysis of ClinicalTrials.gov, we identified studies initiated and completed between January 2010 and December 2023 that contained AI/ML-specific terms in the official title, brief summary, interventions, conditions, detailed descriptions, primary outcomes, or keywords. For 842 completed studies, we searched PubMed and Scopus for publications containing study identifiers and AI/ML-specific terms in relevant fields, such as the title, abstract, and keywords. We calculated disclosure rates within 3 years of study completion and median times to disclosure—from the “primary completion date” to the “results first posted date” on ClinicalTrials.gov or the earliest date of journal publication.

**Results:**

When restricted to studies completed before 2021, ensuring at least 3 years of follow-up in which to report results, 7.0% (22/316) disclosed results on ClinicalTrials.gov, 16.5% (52/316) in journal publications, and 20.6% (65/316) through either route within 3 years of completion. Higher disclosure rates were observed for trials: 11.0% (15/136) on ClinicalTrials.gov, 25.0% (34/136) in journal publications, and 30.1% (41/136) through either route. Randomized controlled trials had even higher disclosure rates: 12.2% (9/74) on ClinicalTrials.gov, 31.1% (23/74) in journal publications, and 36.5% (27/74) through either route. Nevertheless, most study findings (79.4%; 251/316) remained undisclosed 3 years after study completion. Trials using randomization (vs nonrandomized) or masking (vs open label) had higher disclosure rates and shorter times to disclosure. Most trials (85%; 305/357) had sample sizes of ≤1000, yet larger trials (n>1000) had higher publication rates (30.8%; 16/52) than smaller trials (n≤1000) (17.4%; 53/305). Hospitals (12.4%; 42/340), academia (15.1%; 39/259), and industry (13.7%; 20/146) published the most. High-income countries accounted for 82.4% (89/108) of all published studies. Of studies with disclosed results, the median times to report through ClinicalTrials.gov and in journal publications were 505 days (IQR 399-676) and 407 days (IQR 257-674), respectively. Open-label trials were common (60%; 214/357). Single-center designs were prevalent in both trials (83.3%; 290/348) and observational studies (82.3%; 377/458).

**Conclusions:**

For nearly 80% of completed studies, findings remained undisclosed within the 3 years of follow-up, raising questions about the representativeness of publicly available evidence. While methodological rigor was generally associated with higher publication rates, the predominance of single-center designs and high-income countries may limit the generalizability of the results currently accessible.

## Introduction

The number of studies on artificial intelligence/machine learning (AI/ML) has surged in recent years, exceeding prior expectations [1]. The growth of AI/ML research in health care continues to gain momentum, driven by its potential to enable early detection of serious conditions in resource-constrained settings or facilitate timely identification of patient deterioration that might otherwise go unnoticed, to name a few. However, publicly available data are highly heterogeneous, including promotional claims, forward-looking statements, white papers, and preprints. It is often unclear what data underpin the claims made for AI/ML tools, whether regulated, nonregulated, or nonproprietary.

Weak publication records in AI/ML research have been highlighted [2,3], and even when AI/ML research results are available in peer-reviewed journals, they were found to be poorly reported [4,5]; lacking transparency, hindering replicability [6]; involving “spin” and hype by overinflating models’ predictive abilities [7]; or being at high risk of bias [8,9]. These challenges complicate the interpretation of published results and make it difficult to determine whether new findings ultimately provide tangible benefits to patients.

As AI/ML research continues to proliferate, so do systematic reviews and meta-analyses, which presuppose “all relevant evidence” as their foundation. However, this principle is undermined when underreporting is pervasive. Underreporting can lead to biased effect estimates and compromise the diagnostic or prognostic performance of AI/ML, on which clinical decision-making depends. After all, systematic reviews can only reflect the results accessible—a portion of the total research conducted. Lock and Wells [10] have deemed underreporting of research a form of both scientific and ethical misconduct, as it not only leads to biased and imprecise effect estimates but also breaches implied contracts with study participants, who contribute with the expectation of advancing knowledge [11].

The underreporting of clinical trial results has been well documented, long before the emergence of AI/ML. For example, a 2003 study in Spain revealed that fewer than one-third (31%; 38/123) of clinical trials approved by an ethics committee at a major hospital had results published in peer-reviewed journals within 3 years of study completion [12]. Numerous studies using the ClinicalTrials.gov database have provided further evidence since [13-19]. This enables us to compare the disclosure rates of AI/ML trials with those previously reported for non-AI/ML trials registered on ClinicalTrials.gov.

Finally, AI’s impact on health equity is also extensively debated. Some argue that access to the very factors driving AI in health care, such as electronic health records and computing power, may exacerbate existing health care disparities and perpetuate inequities in who benefits most from AI [20]. Certain population groups—demographic, geographic, or economic —can be disproportionately overrepresented in clinical AI/ML research.

This study examines (1) the proportion and patterns of public disclosure of AI/ML research results through ClinicalTrials.gov and peer-reviewed publications indexed in PubMed or Scopus, and (2) whether disclosure rates vary according to key study characteristics, including those related to population representation, such as geographic region and gross national income.

## Methods

### Data Source

We used ClinicalTrials.gov, a trial registry and results database, and sourced data from the Clinical Trials Transformation Initiative Aggregate Analysis of ClinicalTrials.gov (CTTI AACT) [21], which allows open access to the complete set of studies registered in ClinicalTrials.gov, including additional fields that are not readily available in direct exports from ClinicalTrials.gov. The CTTI AACT data dictionary is publicly accessible [22]. A static version of the CTTI AACT database was downloaded for analysis on February 6, 2024, via PostgreSQL, as previously described [23]. We identified studies that initiated and completed between January 2010 and December 2023 and contained AI/ML-specific terms in the official title, detailed description, brief summary, interventions, conditions, primary outcomes, and keywords. These terms could have appeared in studies where AI/ML was used either as an intervention or a method. A total of 842 AI/ML studies were completed by the end of 2023. The search strategies are provided in Multimedia Appendix 1, Table S1. The study flow diagram is provided in Multimedia Appendix 2, Figure S1. Detailed methods on data extraction, including SQL codes, are available in Multimedia Appendix 3.

### AI/ML Publications Linked to ClinicalTrials.gov Identifiers (NCT Numbers)

NCT numbers are unique study identifiers assigned by ClinicalTrials.gov. To identify studies with corresponding journal publications, we searched PubMed and Scopus for these identifiers using each database’s application programming interface (API). In PubMed, we searched for NCT numbers in the titles, abstracts, and “trial registration” fields, while in Scopus, we searched the titles and abstracts. Additionally, we accessed “publication” data on ClinicalTrials.gov using the “reference_type” field in the AACT database. Details on the APIs used for PubMed and Scopus are provided in Multimedia Appendix 3.

Since multiple publications may exist under the same NCT, and not all are related to AI/ML, we included only those that contained AI/ML-specific terms in the title, abstract, keywords, or Medical Subject Headings (MeSH) fields in PubMed. In Scopus, AI/ML terms were searched in the titles and abstracts.

To capture articles likely to be the direct study output, we excluded nonrelevant article types (eg, protocols, reviews, meta-analyses) and those published before primary completion dates. ClinicalTrials.gov defines the primary completion date as the date on which data collection is completed for all primary outcomes [24].

### Main Outcomes and Measures

After identifying eligible articles, we determined the proportion of AI/ML studies that disclosed results within 3 years of the primary completion date, either on ClinicalTrials.gov or in journal publications (the number of studies with disclosed results divided by the number of completed studies). We focused on disclosures via ClinicalTrials.gov or peer-reviewed publications (not preprints), as these are typically searched for systematic reviews and meta-analyses.

For studies with disclosed results, we also calculated the median time from the primary completion date to the disclosure date along with the IQR. The disclosure date was defined as either the “results first posted date” recorded on ClinicalTrials.gov or the date of the earliest journal publication linked to the NCT. If a study had multiple publications, only the earliest publication date was used. Each NCT with at least one publication was counted as disclosed.

All variables were derived from ClinicalTrials.gov. The variable on countries (study location) was categorized according to the World Bank’s gross national income–based classification: low income (≤US $1135), lower-middle income (US $1136 to $4465), upper-middle income (US $4466 to $13,845), or high income (≥US $13,846) [25]. On lead sponsor, those labeled “other” were reassigned into hospital/clinic, academia, industry, government, and nonprofit organization based on lead sponsor names.

Details on the data extraction are provided in Multimedia Appendix 3.

### Statistical Analysis

Data were summarized descriptively: count, percentage, and the median with the IQR. Categorical variables are presented as counts and percentages within 3 years of the primary completion date. Tabulations exclude missing values (ie, n=842 if a variable had no missing values).

### Ethics Approval and Reporting Guidelines

Ethics approval was not required for this study because only publicly available data were analyzed. This study followed the Strengthening the Reporting of Observational Studies in Epidemiology (STROBE) reporting guidelines [26].

## Results

### Overview of Completed Studies on AI/ML

Of 842 completed studies (n=357 interventional; n=485 observational), only 5.5% (46/842) disclosed results on ClinicalTrials.gov, 13.9% (117/842) in journal publications, and 17.7% (149/842) through either route within 3 years of completion (Multimedia Appendix 2, Figure S1).

The disclosure rates were higher among trials only: 10.4% (37/357) on ClinicalTrials.gov, 19.3% (69/357) in journal publications, and 26.1% (93/357) through either route. Rates among randomized controlled trials (RCTs) were even higher: 11.3% (23/203) on ClinicalTrials.gov, 24.6% (50/203) in journal publications, and 32% (65/203) through either route.

Among studies with disclosed results, the median reporting times were 505 days (IQR 399-676) on ClinicalTrials.gov and 407 days (IQR 257-674) in journal publications.

**Figure 1:**
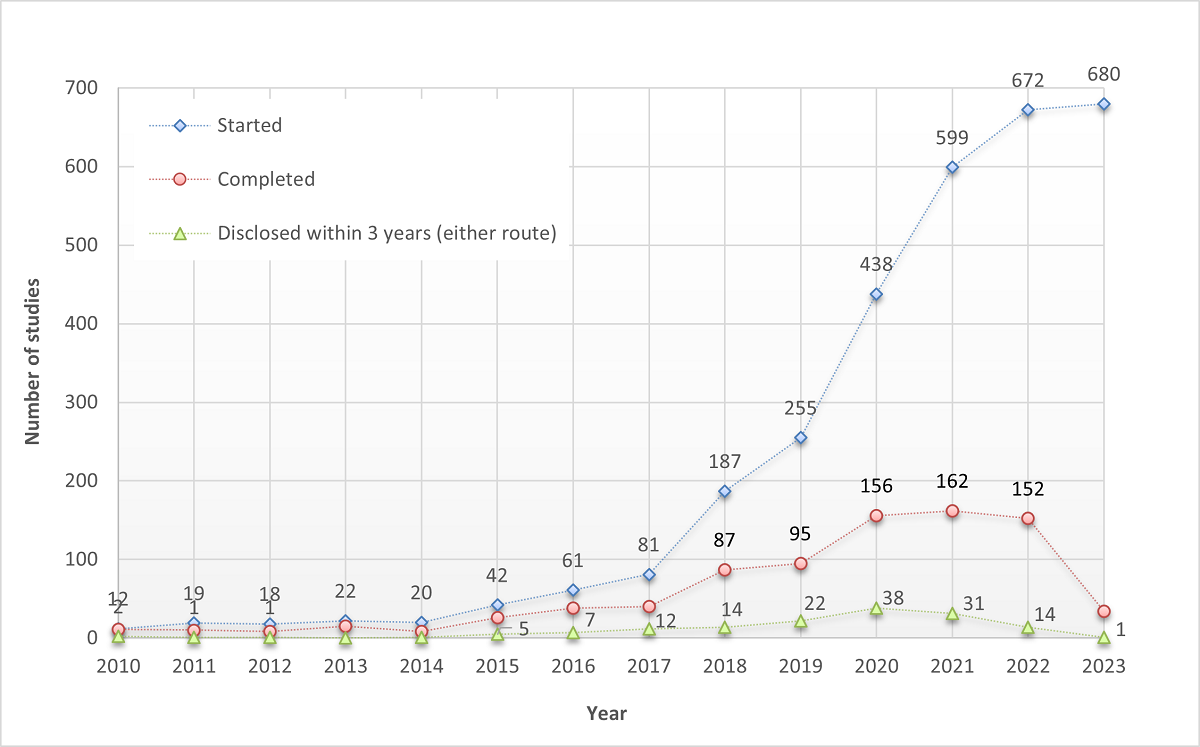
Studies initiated (“started”), studies completed (“completed”), and completed studies with results reported either on ClinicalTrials.gov or in journal publications within 3 years of completion (“disclosed within 3 years”). Despite a surge in new studies each year, the number of studies reporting results through either route remained scarce.

When restricted to studies completed before 2021, ensuring at least 3 years of follow-up in which to report results, 20.6% (65/316) disclosed results through either route within 3 years, and rates were higher among trials (30.1%; 41/136) and RCTs (36.5%; 27/74). Figure 1 shows the trend in studies started each year, completed studies, and studies that reported results within 3 years of study completion. Despite a surge in new studies each year, studies that disclosed results through either route remained scarce.

### Result Disclosure Rates and Time to Disclosure by Study Characteristics

Table 1 shows reporting rates stratified by the primary completion year. The reporting rates on ClinicalTrials.gov remained low over time. However, from 2018 to 2020, there was a modest increase in publication rates within 3 years of completion, and the median time to publication decreased from approximately 2 years (723 days) to 1 year (385 days). The IQR also decreased from 1166 days in 2018 to 279 in 2020. Before 2018, there were too few AI/ML publications to allow meaningful interpretation, and 2021-2023 was not assessable because the 3-year grace period had not passed as of our data cutoff on December 31, 2023.

Table 2 presents disclosure rates and times stratified by various study characteristics.

Disclosure rates and their timing varied by study design and setting (Table 2), as summarized below.

**Table 1:** Results disclosure rates and the time to disclosure, stratified by primary completion year.

Primary completion year	Completed studies, n	CTG^a^ posting ≤3 years, n (%)	Journal publication ≤3 years, n (%)	Time to CTG posting^b^ (days), median (IQR)	Time to journal publication^b^ (days), median (IQR)
2010	1	0 (0)	1 (100)	—^c^	—
2011	2	0 (0)	0 (0)	—	—
2012	3	0 (0)	1 (33)	—	—
2013	3	0 (0)	0 (0)	—	—
2014	7	0 (0)	0 (0)	—	—
2015	9	0 (0)	1 (11)	—	1611 (1100-2122)
2016	20	1 (5)	2 (10)	1026 (938-1113)	487 (415-1049)
2017	25	4 (16)	4 (16)	917 (806-953)	820 (586-1077)
2018	55	4 (7)	5 (9)	538 (512-586)	723 (366-1532)
2019	82	6 (7)	14 (17)	611 (433-821)	681 (339-954)
2020	109	8 (7)	24 (22)	427 (288-526)	385 (274-553)

^a^CTG: ClinicalTrials.gov.

^b^Reflects all studies that reported results (including those disclosed after 3 years of completion).

^c^Not applicable.

**Table 2:** Disclosure rates and the time to disclosure by study characteristics. Tabulations exclude missing values (ie, n=842 if no missing values).

Study characteristics			Completed studies, n		CTG^a^ posting ≤3 years, n (%)		Journal publication ≤3 years, n (%)		Time to CTG posting^b^ (days), median (IQR)		Time to journal publication^b^ (days), median (IQR)
**All completed**			842		46 (5.5)		117 (13.9)		505 (399-676)		407 (257-674)
**Completed before 2021**			316		22 (7.0)		52 (16.5)		574 (445-847)		545 (301-892)
**Study type (n=842)**											
	Inverventional	357		37 (10.4)		69 (19.3)		485 (397-574)		368 (272-624)	
	Observational	485		9 (1.9)		48 (9.9)		722 (526-871)		429 (253-701)	
**Randomization (interventional; n=357)**											
	Randomized	203		23 (11.3)		50 (24.6)		485 (399-565)		357 (273-565)	
	Nonrandomized	154		37 (24)		19 (12.3)		466 (398-593)		549 (262-780)	
**Masking (interventional; n=357)**											
	Open label	214		23 (10.7)		37 (17.3)		485 (397-575)		474 (297-672)	
	Single blind	76		4 (5.3)		16 (21.1)		562 (455-734)		303 (261-405)	
	Double, triple, quadruple blind	67		10 (14.9)		16 (23.9)		486 (363-548)		335 (215-658)	
**Study center (interventional; n=348)**											
	Single center	290		28 (9.7)		55 (19)		495 (399-641)		373 (274-616)	
	Multicenter	58		8 (13.8)		10 (17.2)		443 (404-494)		308 (218-698)	
**Study center (observational; n=458)**											
	Single center	377		8 (2.1)		35 (9.3)		690 (506-834)		427 (252-681)	
	Multicenter	81		0 (0)		10 (12.3)		—^c^		426 (327-529)	
**Enrollment (interventional; n=357)**											
	≤100	181		19 (10.5)		28 (15.5)		427 (397-648)		343 (272-624)	
	101-1000	124		11 (8.9)		25 (20.2)		490 (418-583)		373 (252-560)	
	1001-5000	31		3 (9.7)		10 (32.3)		462 (442-465)		594 (334-743)	
	>5000	21		4 (19)		6 (28.6)		479 (358-537)		413 (279-597)	
**Enrollment (observational; n=485)**											
	≤100	145		4 (2.8)		17 (11.7)		794 (627-863)		289 (231-423)	
	101-1000	206		5 (2.4)		21 (10.2)		638 (526-835)		542 (386-777)	
	1001-5000	82		0 (0)		9 (11)		—		392 (246-459)	
	>5000	52		0 (0)		1 (1.9)		—		1282 (944-1745)	
**Time perspective (observational^d^; n=468)**											
	Prospective	233		6 (2.6)		25 (10.7)		754 (546-892)		386 (246-628)	
	Retrospective	164		3 (1.8)		14 (8.5)		690 (527-787)		457 (362-714)	
	Cross-sectional	71		0 (0)		9 (12.7)		—		427 (333-532)	
**Primary purpose: top 4 (n=221)**											
	Treatment	69		9 (13)		11 (15.9)		505 (467-634)		349 (309-521)	
	Diagnostic	95		7 (7.4)		24 (25.3)		555 (454-671)		430 (282-747)	
	Prevention	29		7 (24.1)		5 (17.2)		433 (322-550)		188 (94-411)	
	Health services research	28		5 (17.9)		8 (28.6)		400 (189-424)		293 (215-565)	
**Lead sponsor: top 5 (n=807)**											
	Hospital	340		4 (1.2)		42 (12.4)		463 (323-540)		407 (274-674)	
	Academia	259		22 (8.5)		39 (15.1)		485 (412-618)		402 (273-624)	
	Industry	146		10 (6.8)		20 (13.7)		651 (318-808)		398 (224-681)	
	Government	18		2 (11.1)		3 (16.7)		720 (605-835)		280 (233-555)	
	Nonprofit organization	44		6 (13.6)		8 (18.2)		536 (458-551)		435 (338-522)	
**Regulation status (n=842)**											
	FDA^e^ regulated	59		14 (23.7)		14 (23.7)		547 (403-707)		467 (233-608)	
	Not FDA regulated	783		32 (4.1)		103 (13.2)		490 (400-662)		397 (272-678)	
**US study site (n=842)**											
	US site	228		35 (15.4)		34 (14.9)		490 (400-611)		440 (296-624)	
	No US site	614		13 (2.1)		83 (13.5)		571 (374-814)		375 (248-677)	
**Study location: region (n=806)**											
	Europe	299		3 (1)		42 (14)		821 (666-966)		375 (251-515)	
	Asia and Pacific	192		4 (2.1)		24 (12.5)		571 (507-612)		411 (264-732)	
	North America	235		33 (14)		37 (15.7)		490 (400-611)		430 (282-638)	
	Middle East	33		3 (9.1)		4 (12.1)		246 (170-540)		200 (158-338)	
	Africa	10		1 (10)		1 (10)		424 (424-424)		577 (577-577)	
	Central and South America	6		0 (0)		0 (0)		—		—	
**Study location: gross national income^f^ (n=775)**											
	High-income countries	613		42 (6.9)		89 (14.5)		505 (399-676)		389 (255-616)	
	Upper-middle-income countries	139		0 (0)		16 (11.5)		—		391 (236-718)	
	Lower-middle-income countries	23		2 (8.7)		3 (13)		490 (457-522)		430 (375-504)	
	Low-income countries	0		0 (0)		0 (0)		—		—	

^a^CTG: ClinicalTrials.gov.

^b^Time to results reporting (posting on CTG or in journal publications) also includes those reported after 3 years.

^c^Not applicable.

^d^Time perspective: data available only for observational studies.

^e^FDA: Food and Drug Administration.

^f^Gross national income–based classification as per the World Bank.

### Interventional Studies (vs Observational)

Trials accounted for 42.4% (357/842) of completed studies; they had higher publication rates (19.3% vs 9.9%) and posted results on ClinicalTrials.gov more often (10.4% vs 1.9%), with shorter times to disclosure than observational studies.

### Randomization

RCTs accounted for 56.9% (203/357) of completed trials and had higher publication rates than non-RCTs (24.6% vs 12.3%). However, non-RCTs posted results on ClinicalTrials.gov more often than RCTs (24% vs 11.3%). RCTs reached publication sooner than non-RCTs (357 vs 549 days).

### Masking

Open-label trials were the most common (59.9%; 214/357), followed by single-blind (21.3%; 76/357) and double-blind or higher (18.8%; 67/357) designs. Single-blind trials (21.1%; 16/76), as well as double-, triple-, or quadruple-blinded trials combined (23.9%; 16/67) had higher publication rates and reached publication sooner than open-label trials (17.3%; 37/214).

### Single Center (vs Multicenter)

Single-center designs dominated both trials (83.3%; 290/348) and observational studies (82.3%; 377/458). The disclosure rates did not vary substantially between single-center and multicenter studies.

### Enrollment

Most trials (85%; 305/357) had sample sizes of ≤1000, yet larger trials (n>1000) had higher publication rates (30.8%; 16/52) than smaller trials (n≤1000) (17.4%; 53/305). However, among observational studies, larger studies (n>1000) had slightly lower publication rates (7.5%; 10/134) than smaller studies (10.8%; 38/351).

### Time Perspective (Observational Studies Only)

Nearly half of observational studies used prospective designs (49.8%; 233/468), followed by retrospective designs (35%; 164/468). Prospective studies had slightly higher publication rates (10.7%; 25/233) and reached publication sooner (386 days) than retrospective studies (8.5%; 14/164; 457 days).

### Lead Sponsor

The majority of studies were sponsored by hospitals (40.4%; 340/842) and academia (30.8%; 259/842). The most published sponsors were hospitals (12.4%; 42/340), academia (15.1%; 39/259), and industry (13.7%; 20/146), but the percentages were slightly higher for studies sponsored by governments (16.7%; 3/18) or nonprofit organizations (18.2%; 8/44), albeit with small event numbers.

### Primary Study Purpose

Diagnostic (31.9%; 95/298) and treatment (23.2%; 69/298) were the most common study purposes. Publication rates were highest for diagnostic (25.3%; 24/95) and health services research (28.6%; 8/28). Prevention studies were more frequently reported on ClinicalTrials.gov (24.1%; 7/29) than in journal publications (17.2%; 5/29). Prevention studies reached publication sooner (188 days) than health services research (293 days), treatment research (349 days), and diagnostic studies (430 days).

### Study Locations (Geographic and Economic)

The majority of completed studies were conducted in Europe (37.1%; 299/806); North America (29.2%; 235/806); or the Asia-Pacific (23.8%; 192/806). When categorized by national income level, most studies were conducted in high-income countries (79%; 613/775), followed by upper-middle-income (18%; 139/775) and lower-middle-income (3%; 23/775) countries, and none in low-income countries. Publication rates were also highest in Europe, North America, Asia, and high-income countries, followed by upper-middle-income countries. Finally, 82.4% (89/108) of studies with corresponding publications were conducted in high-income countries.

### Regulatory Status

Only 7% (59/842) of completed studies were “FDA-regulated,” while 72.9% (614/842) did not involve US sites. FDA-regulated studies had higher publication rates than non-regulated studies (23.7% vs 13.2%). FDA-regulated studies (vs nonregulated) and those with a US site (vs no US site) were more likely to post results on ClinicalTrials.gov.

## Discussion

The proliferation of AI/ML research continues, accompanied by a rise in systematic reviews. However, such reviews can capture only the published portion of all relevant evidence. In this cross-sectional analysis of 842 AI/ML studies completed during 2010-2023, we quantified the extent of results that remain undisclosed years after completion.

### Principal Findings

First, despite a surge in new AI/ML studies each year, most study findings remained undisclosed even 3 years after study completion, which is more lenient than the 2-year grace period used elsewhere [13,14]. Of 842 completed studies, only 17.7% (149/842) disclosed results through either route within 3 years, although rates were somewhat higher among trials (26.1%; 93/357) and RCTs (32%; 65/203). When restricted to studies completed before 2021, ensuring at least 3 years of follow-up in which to report results, 20.6% (65/316) disclosed results through either route within 3 years, and rates were higher among trials (30.1%; 41/136) and RCTs (36.5%; 27/74).

Second, study features of greater methodological or logistical rigor—such as interventional (vs observational), randomization (vs nonrandomized), masking (vs open label), and larger sample sizes (>1000 vs ≤1000) *in trials*—had higher disclosure rates with shorter median reporting times through either dissemination route. However, multicenter status had no discernible impact. Less rigorous features, such as single-center designs (82.8%; 667/806) and open-label trials (60%; 214/357), were prevalent.

Finally, most published studies were from high-income countries (82.4%; 89/108).

### Comparison With Previous Work

#### AI/ML Trials Versus Non-AI/ML Trials Registered on ClinicalTrials.gov

To compare results with those of previous research on trials registered on ClinicalTrials.gov, a trial-only subgroup was used. Among 136 trials completed before 2021, the disclosure rate through either route within 3 years of completion was 30.1% (41/136). This rate was notably lower than that for non-AI/ML settings: oncology trials (60.7%; 7425/12,240 within 2 years) [13], pharmaceutical and biopharmaceutical phase II-IV efficacy trials (25.2%; 3822/15,084 within 1 year) [18], National Institutes of Health–funded trials (46.3%; 294/635 within 30 months) [15], completed trials (45.9%; 311/677 within 2 years) [19], trials by US-based academic medical centers (35.9%; 1560/4347 within 2 years) [14], phase III-IV RCTs of drug interventions (50%; 297/594) [16], and trials approved by a hospital ethics committee in Spain (31%; 38/123 within 3 years). Our 3-year disclosure rate (30.1%; 41/136, or roughly 10% per year) was lower than the 1-year rate for mobile health trials (18.5%; 25/135) [27].

Several factors could explain the lower publication rates. First, for the non-AI/ML trials cited above, registration and result disclosure may have been mandatory (eg, regulated, publicly funded, efficacy trials), unlike much of digital health research [28]. Second, the rarity of efficacy studies in AI/ML research to date may play a role, as exploratory or formative research may be deemed less publishable. Even so, however, summary results (objective data) can still be posted on ClinicalTrials.gov, making otherwise unpublishable results accessible. Third, the lack of reporting standards for AI/ML studies is another factor. However, a range of AI/ML-specific tools now exist, such as the Checklist for Artificial Intelligence in Medical Imaging (CLAIM) 2024 Update [29] and the Consolidated Reporting Guidelines for Machine Learning Modeling Studies [30]. Fourth, preprint servers are widely used in computer science, reflecting the field’s emphasis on speed, openness, and adaptability. If preprints are considered sufficient—particularly when validated through reputable conferences—the urgency for peer-reviewed publication may be lower within the AI community. As a form of sensitivity analysis, we assessed the use of preprints in AI/ML studies registered on ClinicalTrials.gov by searching arXiv, bioRxiv, Research Square, medRxiv, SSRN, and PsyArXiv. We identified only 14 preprints linked to NCT numbers; of these, 6 were subsequently published in journals (already captured in our findings), while the remaining 8 were available only as preprints. That is, among the 693 completed studies with no disclosed results, just 1.2% (8/693) had NCT-matched preprints. Thus, even if preprints were considered as an additional dissemination route, the overall disclosure rate would increase only marginally to 18.6% (157/842), compared to the base case of 17.7% (149/842), which does little to explain the low publication rates.

#### Trial Designs

Previous research in non-AI/ML settings (mostly drugs or biomedical) indicated that trials using randomization, masking, larger sample sizes, or multicenter designs were more likely to be published [13,31,32]. Our findings align with these patterns—except for multicenter status. Although methodological rigor was generally associated with higher publishability, less rigorous features—such as single-center (83.3%; 290/348) or open-label designs (60%; 214/357)—were common among the AI/ML trials. Notably, single-center designs predominated in both trials (83.3%; 290/348) and observational studies (82.3%; 377/458). This pattern was consistent with recent systematic reviews of RCTs on AI, where single-center designs accounted for 62.8% (54/86) [33,34] and 59% (23/39) [34].

#### The Distribution of Studies by Economic Status

According to scoping reviews on AI publications in health care, high-income countries accounted for 73.3% (33/45) of articles published in 2011-2022 [35] as well as 94.5% (240/254) and 93.3% (235/251) of those published in 2019 [20]. In our data, 82.4% (89/108) of the studies with publications from 2010-2023 were from high-income countries, and 14.8% (16/108) were from upper-middle-income countries, most of which were from China (88%; 14/16). That is, high-income countries and China accounted for 95.4% (103/108) of studies with published results. Despite differences in data sources and methodologies, high-income countries were consistently overrepresented across studies.

### Broader Implications of the Study

AI/ML is increasingly being evaluated both as an intervention and as a decision-support tool for clinicians. Yet, in our sample, findings from over 80% of completed studies remained undisclosed even after 3 years, raising concerns about representativeness in systematic reviews or meta-analyses. Reviews that rely on skewed samples of favorable results—excluding null or negative findings, mostly from single-center data—could risk overestimating the effects attributed to AI/ML. This issue may be addressed, in part, by leveraging public registries to improve access to unpublished results. Searching trial registries, such as ClinicalTrials.gov, has been strongly recommended for comprehensive systematic reviews and is mandatory for best-practice Cochrane reviews [36]. This may help reduce publication bias and research waste. To this end, we advocate for broader use of the “results database” feature of public registries and the posting of summary results, even when submission is not mandatory.

The predominance of single-center data and studies from high-income countries has further implications, potentially contributing to existing health inequities. This underscores the need for external validation in diverse populations, especially for models built with single-center data [35]. However, the predominance of single-center designs, along with the shortage or inadequacy of external validation [23,37-39], could be a reflection of what is known as the reproducibility crisis [40-45]. AI researchers face intense pressure to publish quickly, with numerous papers posted daily on arXiv without peer review, and many are reluctant to report failed replications [43]. Unpublished code and sensitivity to training conditions make it difficult, if not impossible, to verify claimed performance [42,46-48]. Notably, papers that fail to replicate are cited at the same rate as those that are successfully replicated, leading to future work being built upon irreproducible results [44,49]. A 2018 study found that only 6% of research presented at top AI conferences explicitly identified research questions being addressed, and just 5% specified hypotheses being tested [40]. Clearly, significant challenges exist even before introducing the added complexities of multicenter studies, which involve substantial clustering (eg, across multiple centers, regions, or countries) and require more rigorous design, analysis, and reporting methods compared to standard prediction model studies [50].

Finally, publishability could improve through the wider adoption of AI/ML-specific reporting guidelines and checklists [51], including those that emphasize critical yet often undocumented details, such as computational reproducibility, data preprocessing, and mitigation of data leakage [41]. Although improved transparency through standardized reporting is the responsibility of authors, guidance such as the REFORMS: Consensus-based Recommendations for Machine-learning-based Science [41] is designed to inform all stakeholders, including readers, about best practices. All the transparency in the field will not suffice unless informed community members critically engage with the key dimensions intrinsic to AI/ML that must be addressed. As preprints gain wider visibility, the role of community literacy will grow increasingly important.

### Limitations

First, to identify relevant publications, we searched for study identifiers (NCT numbers) in the title, abstract, and “trial registration” fields in PubMed, while in Scopus, we searched the title and abstract fields. Some publications may have been missed if the NCT was mentioned outside these fields. However, the International Committee of Medical Journal Editors [52] recommends listing the trial registration number at the end of the abstract, and we assumed compliance with this. We think that this was a reasonable assumption, given that publications mentioning NCTs outside abstracts tended to be cited by others or related in some way rather than being direct research outputs.

Second, we searched for publications only for studies marked “completed.” We may have missed studies with relevant publications if there were delays by sponsors in updating the study status on ClinicalTrials.gov. Nevertheless, this approach also ensured that we captured publications that were likely to be the direct study output. To mitigate this potential misclassification, however, we delayed the data export until February 6, 2024, despite the data cutoff being December 31, 2023. As for publications, we searched PubMed and Scopus on February 12, 2024, allowing 6 weeks after the data cutoff to account for indexing lag time.

Third, information on the study phase may influence the extent of public disclosure of results. However, it was mostly missing (98%; 826/842), and we were unable to use it as a stratifying variable.

### Conclusion

For nearly 80% of completed studies, findings remained undisclosed within the 3 years of follow-up, raising questions about the representativeness of publicly available evidence.

While methodological rigor was generally associated with higher publication rates, the predominance of single-center designs and high-income countries may limit the generalizability of the results that are currently accessible.

## Appendix

Multimedia Appendix 1 Search strategy.

Multimedia Appendix 2 Study flow diagram.

Multimedia Appendix 3 Technical document.

## Abbreviations

AI/ML: artificial intelligence/machine learning
API: application programming interface
CLAIM: Checklist for Artificial Intelligence in Medical Imaging
CTTI AACT: Clinical Trials Transformation Initiative Aggregate Analysis of ClinicalTrials.gov
FDA: Food and Drug Administration
GNI: gross national income
MeSH: Medical Subject Headings
NCT: National Clinical Trial
RCT: randomized controlled trial
STROBE: Strengthening the Reporting of Observational Studies in Epidemiology

## Data Availability

All data generated or analyzed during this study are included in this published article and its supplementary information files.
