# Correction: Public Disclosure of Results From Artificial Intelligence/Machine Learning Research in Health Care: Comprehensive Analysis of ClinicalTrials.gov, PubMed, and Scopus Data (2010-2023)

**DOI:** 10.2196/75554

**Published:** 2025-04-22

**Authors:** Shoko Maru, Ryohei Kuwatsuru, Michael D Matthias, Ross J Simpson Jr

**Affiliations:** 1 Real‑World Evidence and Data Assessment (READS) Graduate School of Medicine Juntendo University Tokyo Japan; 2 Department of Radiology School of Medicine Juntendo University Tokyo Japan; 3 Matthias IT Pty Ltd Brisbane Australia; 4 Division of Cardiology School of Medicine University of North Carolina Chapel Hill Chapel Hill, NC United States

In “Public Disclosure of Results From Artificial Intelligence/Machine Learning Research in Health Care: Comprehensive Analysis of ClinicalTrials.gov, PubMed, and Scopus Data (2010-2023)” (J Med Internet Res 2025;27:e60148), the authors made the following changes.

In the Results section of the Abstract, the sentences:

Of 842 completed studies (n=357 interventional; n=485 observational), 5.5% (46/842) disclosed results on ClinicalTrials.gov, 13.9% (117/842) in journal publications, and 17.7% (149/842) through either route within 3 years of completion. Higher disclosure rates were observed for trials: 10.4% (37/357) on ClinicalTrials.gov, 19.3% (69/357) in journal publications, and 26.1% (93/357) through either route. Randomized controlled trials had even higher disclosure rates: 11.3% (23/203) on ClinicalTrials.gov, 24.6% (50/203) in journal publications, and 32% (65/203) through either route. Nevertheless, most study findings (82.3%; 693/842) remained undisclosed 3 years after study completion.

Have been revised to:

When restricted to studies completed before 2021, ensuring at least 3 years of follow-up in which to report results, 7.0% (22/316) disclosed results on ClinicalTrials.gov, 16.5% (52/316) in journal publications, and 20.6% (65/316) through either route within 3 years of completion. Higher disclosure rates were observed for trials: 11.0% (15/136) on ClinicalTrials.gov, 25.0% (34/136) in journal publications, and 30.1% (41/136) through either route. Randomized controlled trials had even higher disclosure rates: 12.2% (9/74) on ClinicalTrials.gov, 31.1% (23/74) in journal publications, and 36.5% (27/74) through either route. Nevertheless, most study findings (79.4%; 251/316) remained undisclosed 3 years after study completion.

In the Conclusion section of the Abstract and in the Conclusion:

For over 80% of AI/ML studies completed during 2010-2023, study findings remained undisclosed even 3 years after study completion, raising questions about the representativeness of publicly available evidence.

Has been revised to:

For nearly 80% of completed studies, findings remained undisclosed within the 3 years of follow-up, raising questions about the representativeness of publicly available evidence.

In Table 2, the following row was added immediately below “All completed”:

“Completed before 2021”; “316; 22 (7.0); 52 (16.5); 574 (445-847); 545 (301-892)”

We added the following sentence in the Results section (immediately before “Figure 1 shows…”), and in the Discussion section under Principal Findings (at the end of the first paragraph):

When restricted to studies completed before 2021, ensuring at least 3 years of follow-up in which to report results, 20.6% (65/316) disclosed results through either route within 3 years, and rates were higher among trials (30.1%; 41/136) and RCTs (36.5%; 27/74).

In the Results section under Regulatory Status, the sentence:

FDA-regulated studies had higher publication rates than non-RCTs (23.7% vs 13.2%).

Has been revised to:

FDA-regulated studies had higher publication rates than non-regulated studies (23.7% vs 13.2%).

In the Discussion (Comparison with previous work), the sentence:

Among the 357 trials, the disclosure rate through either route within 3 years of completion was 26.1% (93/357).

Has been revised to:

Among 136 trials completed before 2021, the disclosure rate through either route within 3 years of completion was 30.1% (41/136).

In the Discussion (Comparison with previous work) the sentence:

Our 3-year disclosure rate (26.1%; 93/357), equivalent to roughly 8.7% per year, wasalso lower than the 1-year rate for mobile health trials (18.5%; 25/135).

Has been revised to:

Our 3-year disclosure rate (30.1%; 41/136, or roughly 10% per year) was lower than the 1-year rate for mobile health trials (18.5%; 25/135).

The authors have also replaced Multimedia Appendix 2 with file attached below.

The correction will appear in the online version of the paper on the JMIR Publications website, together with the publication of this correction notice. Because this was made after submission to PubMed, PubMed Central, and other full-text repositories, the corrected article has also been resubmitted to those repositories

## Appendix

Multimedia Appendix 2 Study flow diagram.

