# Supplementary material for: Public Disclosure of Results From Artificial Intelligence/Machine Learning Research in Health Care: Comprehensive Analysis of ClinicalTrials.gov, PubMed, and Scopus Data (2010-2023)
Source: J Med Internet Res. 2025 Mar 21;27:e60148. doi: 10.2196/60148 (PMC11971578; doi:10.2196/60148)
Supplement: Multimedia Appendix 1 [file jmir_v27i1e60148_app1.docx]

**Appendix Table 1: Search strategy.**

| Date searched | 6 February 2024 |
| --- | --- |
| Source data | Clinical Trials Transformation Initiative \|  Aggregate Analysis of ClinicalTrials.gov (AACT) database |
| Table name | STUDIES to retrieve relevant studies |
| Table columns searched | official title, brief title, brief summary, interventions, detailed descriptions, primary outcome, or keywords |
| Period | Start date: from 1-Jan-2010 to 31-Dec-2023 |
| Searched data fields | STUDIES.official_title, DETAILED_DESCRIPTIONS.description, ALL_INTERVENTIONS.names, ALL_KEYWORDS.names, ALL_BROWSE_CONDITIONS.names, BRIEF_SUMMARIES.description, DESIGN_OUTCOMES.measure and DESIGN_OUTCOMES.description (where ‘design_outcome”=primary outcome) |

| Text search - combined with OR |
| --- |
| ai-based  artificial intelligence  artificial neural network*  augmented intelligence  bayes* network*  classification tree*  convolutional neural network*  deep learning  deep neural network*  deep reinforcement learning  elastic net  generative adversarial network*  gradient boosting  k nearest neighb*  machine learning  multilayer perceptron*  naïve bayes or naïve bayes  natural language processing*  random forest  recurrent neural network*  regression tree  reinforcement learning  supervised learning  support vector machine*  unsupervised learning  XGBoost |

The asterisk (*) denotes a wildcard character.
