# Supplementary material for: Public Disclosure of Results From Artificial Intelligence/Machine Learning Research in Health Care: Comprehensive Analysis of ClinicalTrials.gov, PubMed, and Scopus Data (2010-2023)
Source: J Med Internet Res. 2025 Mar 21;27:e60148. doi: 10.2196/60148 (PMC11971578; doi:10.2196/60148)
Supplement: Multimedia Appendix 2 [file jmir_v27i1e60148_app2.docx]

Appendix Figure 1: Study flow diagram.

CTG: ClinicalTrials.gov. NCT: National Clinical Trial. PCD: primary completion date.
Data downloaded on 6FEB2024 (last updated on 6FEB2024).
^a^ Examples: studies where AI/ML terms were used only to refer to previous research or as forward-looking statements.
^b^149 includes studies with both posted and published results (n=14)

Clinical Trials Transformation Initiative

Aggregate Analysis of ClinicalTrials.gov database:

Studies identified by AI/ML search terms

that started during 1JAN2010 to 31DEC2023

(n=3378)

272 studies excluded:

- 265 Not AI/ML-related (only ‘keyword’ hit with no AI/ML-terms used to describe research, or used but unrelated to the study)^a^
- 2 Systematic review/meta-analysis
- 5 ‘AI’ was part of org/product/study name

3106 AI/ML studies included

Studies with NCT-matched publications indexed in PubMed or Scopus published after PCD

(n=186/842)

2264 not completed:

1694 Active

95 Stopped

475 Unknown

Result posted on Clinicaltrials.gov

(n=47/842)

842 completed

And published within 3 years after PCD (n=117/842)

When restricted to studies completed before 2021(n=52/316)

And includes AI/ML terms in the title/abstract, keywords

(n=128/842)

And posted within 3 years after PCD (n=46/842)

Studies that reported ***either*** on ClinicalTrials.gov ***or*** in journal publication within 3 years after PCD (n=149/842)^b^

When restricted to studies completed before 2021 (n=65/316)
