# Supplementary material for: Public Disclosure of Results From Artificial Intelligence/Machine Learning Research in Health Care: Comprehensive Analysis of ClinicalTrials.gov, PubMed, and Scopus Data (2010-2023)
Source: J Med Internet Res. 2025 Mar 21;27:e60148. doi: 10.2196/60148 (PMC11971578; doi:10.2196/60148)
Supplement: Multimedia Appendix 3 [file jmir_v27i1e60148_app3.pdf]

Online Supplementary File - Maru et al 2025

Contents

Overview of data extraction ... 2

Initial set of AI/ML studies from ClinicalTrials.gov ① ... 4

Linking Studies to Published Results ... 5

Searching ClinicalTrials.gov ② ... 5

Searching PubMed ③ ... 6

Searching Scopus ④ ... 7

Creating the *all_pm_ids_set* ⑤ ... 9

Combining the *pm_id* sets and extracting Publication Metadata. ... 9

Retrieving Publication Metadata using PubMed "esummary" API. ... 10

Rule to find earliest Publication Date from esummary API (*PubDate*) ... 11

Retrieving Publication MeSH and Keyword values ... 11

Manually Retrieving Metadata for remanent Scopus publications ... 12

Calculating *SearchKeysFound* ⑥ ... 13

Merging data to form the *full_data_set* ⑦ ... 14

Filtering to form the two output datasets ⑧ ... 15

Abbreviations ... 16

Appendix 1. SQL to Retrieve *nct_id*, *pm_id* links from AACT. ... 17

Background on STUDY_REFERENCES table in AACT database. ... 17

SQL to extract nct_id and pm_id links: ... 18

Appendix 2. VBA - Calculating *SearchKeysFound* ... 18

Appendix 3. VBA for PubMed and Scopus APIs ... 21

VBA "Step 3 PubMedApi.bas" module ... 21

VBA "Step 4 ScopusApi.bas" module ... 21

vba_tools.bas module ... 22

Appendix 4. Python Program to retrieve PubMed MeSH and Keyword attributes ... 22

Overview of data extraction

To evaluate the publication trends and time-to-publication among clinical studies involving artificial intelligence/machine learning (AI/ML) registered on ClinicalTrials.gov, we conducted a multi-step data extraction and integration process. The key objectives were:

1) Identify AI/ML-related studies: We filtered clinical trials registered from January 1, 2010, to December 31, 2023, using relevant AI/ML keywords across study titles, descriptions, and other metadata fields in ClinicalTrials.gov.
2) Link studies to peer-reviewed publications: We cross-referenced the identified studies with publication records in PubMed and Scopus databases using unique study identifiers (*nct_ids*).
3) Analyse publication timelines: We calculated the time from the *primary_completion_date* of each study to its first peer-reviewed publication date (*publicationDate*).

Data extraction was performed across three public databases:

1) **ClinicalTrials.gov**: an SQL query was executed against the Aggregate Analysis of ClinicalTrials.gov (AACT) database[1].
2) **PubMed**: We utilized the National Institutes of Health (NIH) "esearch" API to retrieve publication records[2].
3) **Scopus**: The Elsevier/Scopus API was employed for additional searches[3].

Data from each source was merged to form comprehensive datasets, which were then filtered and analysed based on study completion status, AI/ML relevance, and time-to-publication criteria.

Metadata describing each publication was also downloaded from PubMed and Scopus.

(See Figure 1 for overview dataflow diagram of the full process)

---

[1] Maru et al, 2024; doi:10.2196/57750

[2] PubMed API Docs: https://www.ncbi.nlm.nih.gov/books/NBK25497/

[3] Scopus API Docs: https://dev.elsevier.com/guides/Scopus%20API%20Guide_V1_20230907.pdf

Figure 1. *Overview of Data Extraction and merging processes to produce the full set.*

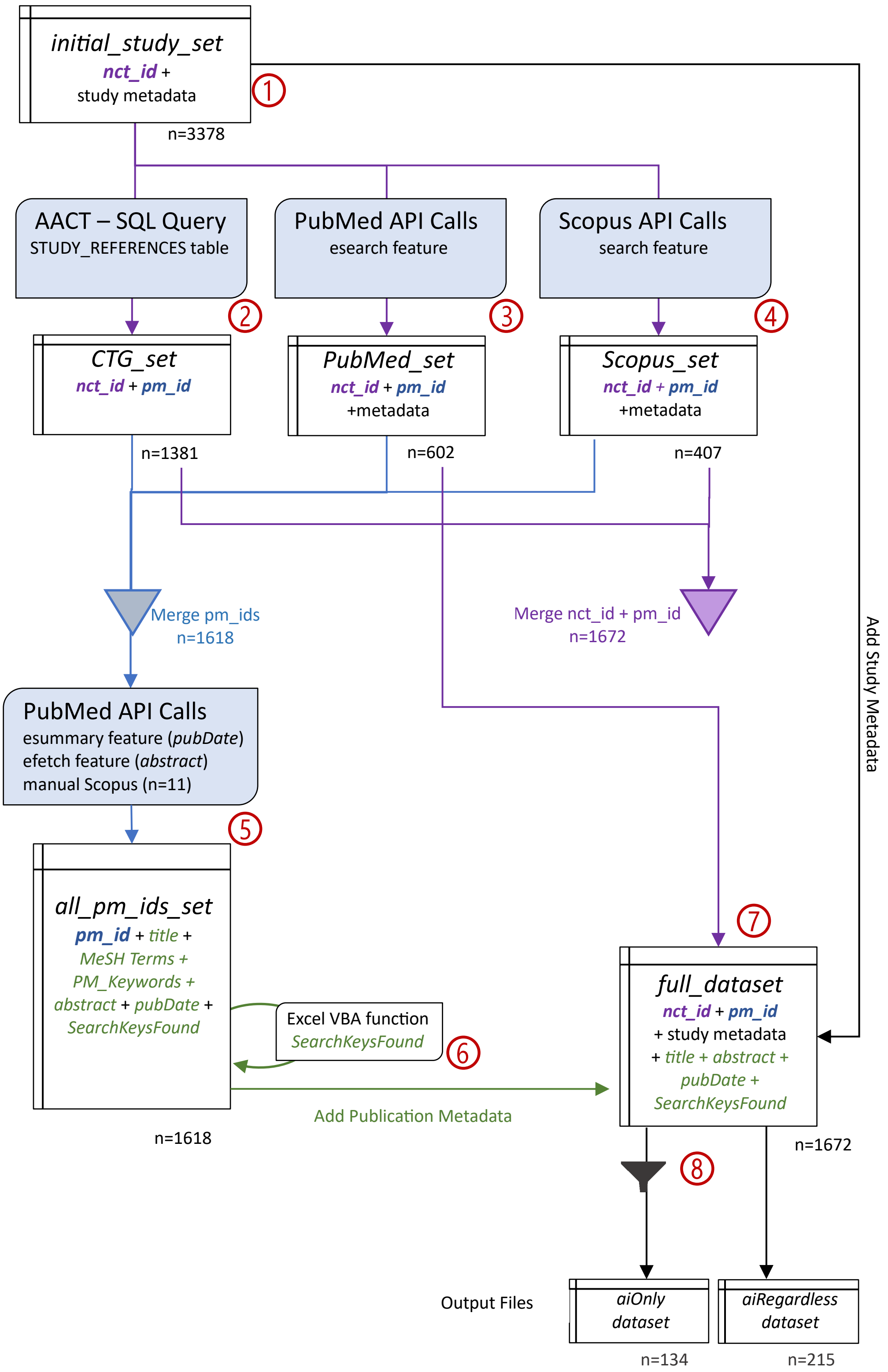

In this document:

nct_id = National Clinical Trial Numbers assigned by AACT

pm_id = document identification numbers assigned by PubMed.

Initial set of AI/ML studies from ClinicalTrials.gov ①

As reported in previous work [4], an SQL was run against the STUDIES table [5], within the AACT reporting database. A filtered set of studies was produced:

(initial_study_set) = list of all studies where

- Filter: *start_date* was registered between 1-Jan-2010 and 31-Dec-2023, and
- Filter: *title*, *description*, *interventions*, *keywords*, *browse_conditions*, *brief_summaries*, *design_outcomes* and/or *design_measures* fields contained search terms related to AI/ML.

**SQL + Manual Process**	Extract Date 2024-02-06	
SQL File:	"Step 1 Set from Previous Paper.sql"	
Input File:	"SQL 1 - MainSet Data.xlsx" (from previous work)	n=3378
Output Sheet:	**"Step_Results.xlsx!Step 1 initial_study_set"**	n=3378

Output Columns:

nct_id	Unique identifier for each Study as defined by AACT
overall_status	Status of the Study (From AACT)
start_yr	Year study commenced (From AACT)
start_date	Date study commenced (From AACT)
primary_completion_date	Primary Date of Completion. (From AACT)
completion_date	Date of Completion. (From AACT, may be an anticipated future date)
last_update_posted_date	Date AACT records were last entered/updated.
FAS	1=Manually determined to be Included in Full Analysis Set from previous work [6]

[4] Maru et al, 2024; doi:10.2196/57750, Supplementary Appendix provides details of the method and SQL code. For convenience the SQL has been replicated here in file: "Step 1 Set from previous paper.sql".

[5] AACT Data Dictionary: https://aact.ctti-clinicaltrials.org/data_dictionary. Search for "STUDIES" table.

[6] Maru et al, 2024; doi:10.2196/57750

Table 1. *Summary of Step 1; List of nct_ids and metadata from ClinicalTrials.gov*

Linking Studies to Published Results

For each AI/ML study identified from ClinicalTrials.gov *(initial_study_set)*, we sought to link associated peer-reviewed publications by searching within ClinicalTrials.gov, PubMed, and Scopus. The linking process involved three key methods to maximize identification accuracy and coverage.

Searching ClinicalTrials.gov ②

The AACT database maintains a table (STUDY_REFERENCES), which links studies *(nct_ids)* to corresponding publications within PubMed *(pm_ids)*. We ran an SQL [7] on the STUDY_REFERENCES table to extract all such links, to form the dataset:

(CTG_set) =

- Filter: *nct_id* matched in our *initial_study_set* and
- Filter: *reference_type* was either "result" or "derived".

SQL Program:	"Step 2 STUDY_REFERENCES extract.sql"	Extract Date 2024-02-06
Input Sheet:	**"Step_Results.xlsx!Step 1 initial_study_set"**	n=3378
Output Sheet:	**"Step_Results.xlsx!Step 2 CTG Set"**	n=1381

Output Columns:

nct_id	Study ID from STUDY_REFERENCES.nct_id column
pmid	Publication ID from STUDY_REFERENCES.pmid column.
reference_type	Type of publication. From STUDY_REFERENCES.reference_type<br>Either "result" or "derived"

Table 2. *Summary of Step 2; Extracting Study+Publication pairs from AACT.STUDY_REFERENCES table*

Note 1: 409 of 3378 studies had links to publications (i.e. 409 unique *nct_ids*).

Note 2: Duplicate *nct_id* and duplicate *pmid* are valid, but the combination of both is unique.

[7] Appendix 1. Provides the SQL code used.

Searching PubMed ③

To identify publications not captured within ClinicalTrials.gov, we queried the PubMed database using the NIH “esearch” API[8]. The API was used to search for *nct_ids* from the *initial_study_set*, across all of PubMed’s indexed fields.

- 602 *nct_id*+*pm_id* pairs linking studies to publications were found.
- 278 pairs were “New Yields”, i.e. links found in PubMed, but not ClinicalTrials.gov

VBA Module File:	“Step 3 PubMedAPI.bas” Extract Date 2024-02-09
Starting Function:	get_PM_batch_from_nct_list()
API Calls:	https://eutils.ncbi.nlm.nih.gov/entrez/eutils/esearch.fcgi?db=pubmed&term=NCT123456789
Input Sheet:	**“Step_Results.xlsx!Step 1 initial_study_set”** n=3378
Output Sheet:	**“Step_Results.xlsx!Step 3 PubMed set”** n=602

Output Columns:

nct_id	Study ID
pubmed_id	Publication ID
reference_type	“NewPMAPI” (literal text)

Table 3. *Summary of Step 3; Extracting Study+Publication pairs from PubMed using API*

See Appendix 3 in this document for a description of the VBA code used in this extract.

[8] e.g. https://eutils.ncbi.nlm.nih.gov/entrez/eutils/esearch.fcgi?db=pubmed&term=NCT123456789
See: https://www.ncbi.nlm.nih.gov/books/NBK25500/#chapter1.Searching_a_Database for NIH reference

Searching Scopus ④

Finally, we searched Scopus, using NCT identifiers, to find any publications that may have been registered outside the ClinicalTrials.gov and PubMed systems. The Elsevier/Scopus API [9], was used to search the *abstract* and *title* fields in Scopus for *nct_ids*. Of the 407 matches found returned, 11 publications had not been found in the previous searches and not indexed to PubMed. These were manually added as new yields with Scopus ID (*eid*).

The majority of Scopus publications (311 *nct_ids* of 407 found by search) returned a *pm_id* to the corresponding publication in PubMed. Where a *pm_id* was not found, we reused the PubMed esearch API (see Step 3) using *doi* as the search key, and obtained a further 83 *pm_ids*. The remaining 13 rows (11 unique) from the extract contained Scopus document IDs *eid* which were used to manually look up the metadata in the Scopus system.

[9] e.g. https://api.elsevier.com/content/search/scopus?query=title-abs(NCT12345678)&apiKey=xxxyyy

VBA Module File:	"Step 4 ScopusAPI.bas"	Extract Date 2024-02-09
Start Function:	get_Scopus_batch_from_nct_list ()	
API Calls:	https://api.elsevier.com/content/search/scopus?query=title-abs(NCT12345678)&apiKey=xxxyyyy<br>https://eutils.ncbi.nlm.nih.gov/entrez/eutils/esearch.fcgi?db=pubmed&term=NCT123456789	
Input Sheet:	**"Step_Results.xlsx!Step 1 initial_study_set"**	
Output Sheet:	**"Step_Results.xlsx!Step 4 Scopus set"**	n=407

Output Columns:

nct_id	Study ID
pubmed_id	Either:<br>*pm_id* if the PubMed ID was found in Scopus API XML or<br>*eid* if not found in XML
reference_type	Result of Scopus API lookup process.<br>"Scopus: PM_id found" if *pm_id* was found in XML (n=311)<br>"Successful DOI Lookup in PubMed" if *doi* was successfully used to find *pm_id* in PubMed API (n=83)<br>"Failed DOI Lookup in PubMed" if neither *pm_id* nor *doi* found. (n=13)
doi	Digital Object Identifier. Unique document id, agnostic to the database it resides on.<br>Value from Scopus API - XML node <prism:doi>
eid	Scopus document ID number.<br>Value from Scopus API - XML node <eid>
coverDate	Date published in SCOPUS<br>Value from Scopus API - XML node <prism:coverDate>
title	Scopus Title XML node <dc:title>

Table 4. *Summary of Step 4; Extracting Study+Publication pairs from Scopus using API*

Creating the *all_pm_ids_set* ⑤

Combining the *pm_id* sets and extracting Publication Metadata.

The sets (CTG_set, PubMed_set and Scopus_set) were combined to form a list of 1618 unique *pm_ids* / *eids*. To this, we added corresponding publication date (*pubDate)*, *title*, and *abstract* fields from PubMed using further API calls to the "esummary" and "efetch" features. The resulting *all_pm_ids_set* had the following structure:

The three sets of *pm_ids* from steps 2, 3 and 4 were combined in Excel to form a single list of unique values.

Manual Excel Process	Extract Date 2024-02-09
Input Sheets:	**"Step_Results.xlsx!Step 2 CTG_Set"**<br>**"Step_Results.xlsx!Step 3 PubMed_Set"**<br>**"Step_Results.xlsx!Step 4 Scopus_Set"**
Output Sheet:	**"Step_Results.xlsx! Step 5a all_pm_id_Set"** n=1618

Output Columns:

pm_id	Unique<br>• PubMed identifier (n=1607) or<br>• Scopus Identifier *eid* (n=11) [10]
source	From *reference_type* column in source data file.<br>Where *pm_id* appeared in multiple files, *reference_type* was selected with the following priority:<br>"result", "derived",<br>"NewPMAPI",<br>"Scopus: PM_id found", "Successful DOI Lookup in PubMed", "Failed DOI Lookup in PubMed"

Table 5. Summary of Process for combining all PubMed Ids

[10] These documents were cases where Scopus database had a publication related to a study, but there was no corresponding publication in PubMed, hence the *pm_id* was missing. *eid* was used instead.

Retrieving Publication Metadata using PubMed “esummary” API.

The PubMed “esummary” and “efetch” API features were used to obtain the following metadata for all *pm_ids* in the *all_pm_ids_set*:

VBA Module File:	“Step 3 PubMedAPI.bas” Extract Date 2024-02-09
Start Function:	main2_Date_and_Abstracts ()
API Calls:	https://eutils.ncbi.nlm.nih.gov/entrez/eutils/esummary.fcgi?db=pubmed&version=2.0&id=pm_id<br>https://eutils.ncbi.nlm.nih.gov/entrez/eutils/efetch.fcgi?db=pubmed&rettype=abstract&retmode=text&id=pm_id
Input Sheet:	**“Step_Results.xlsx! Step 5a all_pm_id_set”** n=1607<br>Filtered where *source* <> “Failed DOI Lookup in PubMed”
Output Sheet:	**“Step_Results.xlsx!Step 5c all_PM_id_lookups”** n=1607

Output Columns:

pm_id	From Input Sheet
source	From Input Sheet, Source column
SearchKeysFound	Calculated from VBA *SearchKeysFound()* function (see rules below)
pubDate	Earliest date that is registered in PubMed for the publication (see rules in section below)<br>See VBA function getEarliestDate()
title	Extract document *title* directly from “//Title” node.<br>See function writeTitle()
abstract	The Abstract was obtained using the “efetch” API feature.<br>See function write_PubMed_abstract()
url	URL used to call the PubMed API
doi	Extract document’s last author directly from "// ArticleId[IdType='doi']/Value" node.<br>See function writeNode()

Table 6. *Summary of Step 5; Extracting Metadata using PubMed esummary API*

An Excel VBA program (see function: main2_Date_and_Abstracts, Appendix 3) was written to iterate through the *all_pm_ids_set*. The metadata was saved into the Excel Table as it progressed. Records where *pm_id* contained a Scopus *eid* were skipped by this process (n=11).

Rule to find earliest Publication Date from esummary API (*PubDate*)

PubMed stores various timestamps for different stages of the publication process. To identify the earliest date per record, the program searched for timestamps in the XML using the following rules:

1) Look in the "//PubMedPubDate" node for the 'pubmed' or 'entrez' dates.
   If found, the earliest of these two dates is chosen.
2) If "//PubMedPubDate" node not found,
   Search for the "//EPubDate" node.
3) If "//EPubDate" node not found,
   Search for the "//PubDate" node.
4) Otherwise return the date 3000-01-01, which signifies no date found in the XML.
   (This did not occur in any records retrieved)

Retrieving Publication MeSH and Keyword values

A python program was written subsequent to the Excel extracts to retrieve all MeSH and Keyword values for each publication. (See Appendix 4 for code and details)
The results were merged into spreadsheet Step_Results.xlsx!Step 5c all_PM_id_lookups columns "MeSH Terms" and "PM_Keywords".

Python File:	"fetch_pubmed_xml.py" Extract Date 2024-12-20
API Calls:	https://eutils.ncbi.nlm.nih.gov/entrez/eutils/efetch.fcgi?db=pubmed&id={pmid}&retmode=xml
Input Sheet:	**"Step_Results.xlsx! Step 5a all_pm_id_set"** n=1607<br>Filtered where *source* <> "Failed DOI Lookup in PubMed"
Output Sheet:	**"Step_Results.xlsx!Step 5b all_PM_id_mesh"** n=1607

Output Columns:

pm_id	From Input Sheet
MeSH Terms	All MeSH terms assigned to the publication's XML record, from the "//MeshHeading/DescriptorName" nodes.
PM_Keywords	A Keyword terms from the publication's "//Keyword" nodes.
SearchKeysFound	Re-calculated using VBA *SearchKeysFound()* function (see rules below)
firstAuthor	Extract document's first author directly from "//SortFirstAuthor" node.<br>See function writeNode()

lastAuthor	Extract document's last author directly from "//LastAuthor" node.<br>See function writeNode()

Manually Retrieving Metadata for remanent Scopus publications

The 11 extra publications found in Scopus were manually entered by copying data from Scopus web pages.   as the *publication_date*.

Manual Excel Process	Extract Date 2024-02-12
Input File:	"Step_Results.xlsx!Step 4 Scopus set" n=11<br>Filtered where *reference_type* = "Failed DOI Lookup in PubMed"
Scopus Page:	https://www.scopus.com/record/display.uri?eid=*eid*
Output Sheet:	Appended to n=1618<br>**"Step_Results.xlsx!Step 5c all_PM_id_lookups"**

Output Columns:

pm_id	*eid* from Input File
source	"Scopus: Manual"
SearchKeysFound	Were AI/ML search keys found in *title, abstract, MeSH Headings* and/or *keywords.*<br>See rule below.
pubDate	*coverDate* from Scopus web site
title	*title* from input file
abstract	Abstract manually copied from Scopus site.
url	URL used to call the Scopus site.

Table 7. *Adding Scopus Publication metadata and AI/ML Keyword search*

Calculating *SearchKeysFound* ⑥

This field was calculated to determine which AI/ML terms were contained within the publication, if any. An Excel User Defined function [11] was written to implement keyword search using Regular Expression matching. Our previous work [12] defined the following search keys for AI/ML:

> *ai-based, artificial intelligence, augmented intelligence, deep learning, convolutional neural network, deep neural network, artificial neural network, recurrent neural network, generative adversarial network, deep reinforcement learning, machine learning, bayesian network, classification tree, elastic net, gradient boosting, xgboost, k nearest neighbour, multilayer perceptron, support vector machine, natural language processing, naive bayes, random forest, regression tree, reinforcement learning, supervised learning, unsupervised learning*

SearchKeysFound =

- A text list of all the AI/ML related search keys that were found within the *title, abstract, MeSH Headings* and/or *keywords* fields for the publication.
- If multiple search keys were found, all found keys are returned, separated by "|" character.

If no matches were found, "No keys found!" text was returned.

An Excel User Defined function was written to implement keyword search using Regular Expression matching.
See function searchForKeyPhrases() in "Module1" for the code.

[11] See function searchForKeyPhrases() in Appendix 2 for the code.

[12] Maru et al, 2024; doi:10.2196/57750

Merging data to form the *full_data_set* ⑦

The three data sets (CTG_set, PubMed_set and Scopus_set) were combined to form a list of 1672 unique *nct_id+pm_id* or *nct_id* + *eid* pairs. The metadata from the *all_pm_ids_set* was merged to form the *full_data_set*.

Manual Excel Process	Extract Date 2024-02-14
Input Sheets:	"Step_Results.xlsx!**Step 1 initial_study_set**"" "Step_Results.xlsx!**Step 5c all_PM_id_lookups**"
Output Sheet:	"Step_Results.xlsx!**Step 7 full_dataset**" n=1672

Output Columns:

nct_id	*Unique*	List of unique *nct_id* and *pm_id* pairs.
pm_id		
FAS		1 = Study is included in final dataset
overall_status		Lookup from Input Sheet 1, *overall_status* column from AACT
source		Lookup from Input Sheet 5c, *source* column
AIML_terms_used		Lookup from Input Sheet 5c, *SearchKeysFound* column
Prim_Compdate		Lookup from Input Sheet 1, *primary_completion_date* from AACT
publicationDate		Lookup from Input Sheet 5c, *PubDate* column
PrimComp Date 2 Pub		Calculated =publication Date – *prim_compDate* in days. Note: can be negative, especially if *prim_compDate* > 2024-01-01 (n=1261)
PubDate aiRegardless		PublicationDate if "aiRegardless" filter condition met, otherwise 0. (See filter conditions in Step 8 below)
EarliestFlag aiRegardless		"Earliest" if "aiRegardless" filter condition met, otherwise null. (See filter conditions in Step 8 below)
PubDate aiOnly		PublicationDate if "aiOnly" filter condition met, otherwise 0. (See filter conditions in Step 8 below)
EarliestFlag aiOnly		"Earliest" if "aiOnly" filter condition met, otherwise null. (See filter conditions in Step 8 below)

Table 8. *Summary of Combined nct_id, pm_id and publication metadata.*

Filtering to form the two output datasets ⑧

Two filtering scenarios were examined, depending upon whether AI/ML search terms were present in the publication's *abstract* and/or *title* fields:

- *aiOnly_dataset* included only publications where *SearchKeysFound* <> 'No Keys Found', i.e. AI/ML keywords were found in the title or abstract of the publication.
- *aiRegardless_dataset* included publications regardless of the value in *SearchKeysFound*.

The following filters were applied both datasets to include rows where :

- *overall_status* = 'Completed' and
- *prim_compDate* <= '2023-12-31' and
- *prim_compDate* < *publicationDate*
- If more than one publication was released subsequent to primary completion date, we selected the publication with the earliest *pubDate*.

Output files aiRegardless.csv and aiOnly.csv are available with this documentation.

Manual Excel Process	Extract Date 2024-02-14	
Input Sheet:	"Step_Results.xlsx!**Step 7 full_dataset"**	
Output Sheets:	"Step_Results.xlsx!**Step 8 aiOnly output"**<br>"Step_Results.xlsx!**Step 8 aiRegardless output"**	n=134<br>n=215

Output Columns:

nct_id	*Unique*	Lists of *nct_id* and *pm_id* from input files Step 2, 3 & 4 were combined to produce a list of unique pairs across all 3 data inputs.
pm_id		
status		*overall_status* from AACT.studies
source		Identifies where the *nct_id + pm_id* pair was found.
AIML_terms_used		Lookup from Input Set 5c, *SearchKeysFound* column
Prim_Compdate		Lookup from Input Set 1, *primary_completion_date* column
publicationDate		Lookup from Input Set 5c, *PubDate* column
PrimComp Date 2 Pub		Calculated =publication Date – *prim_compDate* in days.
FAS		1 = Study is included in initial dataset.

firstAuthor	Lookup from set 5c, *firstAuthor* column (for aiOnly file)
finalAuthor	Lookup from set 5c, *finalAuthor* column (for aiOnly file)
doi	Lookup from set 5c, *doi* column (for aiOnly file)
title	Lookup from set 5c, *title* column (for aiOnly file)

Abbreviations

AACT	Aggregate Analysis of ClinicalTrials.gov.<br>A daily copy of the data in ClinicalTrials.gov. to a Postgres reporting database.
AI/ML	Artificial Intelligence/Machine Learning
API	Application Programming Interface.<br>A standard mechanism to extract data from a system (such as PubMed, Scopus)<br>Often implemented by presenting some data to a web service (i.e. a web address), which then returns data in a structured form.
doi	Digital Object Identifier.<br>A unique identifier assigned by International DOI Foundation.<br>Provides a consistent identification of a document, agnostic to the system it resides on.
eid	Electronic Identifier.<br>Used by Scopus system to track documents within Scopus.
nct_id	National Clinical Trial Identifier.<br>Unique number that identifies a study in ClinicalTrials.gov
NIH	the National Institutes of Health
pm_id, pmid	PubMed Identifier.<br>Unique number assigned by PubMed system.
XML	Extensible Markup Language<br>A standard and flexibly way to transmit and store data in human and computer readable form. Often used to return data from an API.

Appendix 1. SQL to Retrieve *nct_id*, *pm_id* links from AACT.

Background on STUDY_REFERENCES table in AACT database.

Within AACT, this table links an individual study to one (or many) publications in the PubMed system.

AACT documentation [13] describes this table as:

> Citations to publications related to the study protocol and/or results. Includes PubMed Unique Identifier (PMID) and/or full bibliographic citation.

The columns are populated from various nodes in the PubMed API response XML.

The database columns are:

Column Name	Use
ID	Unique id for table row added by CT.gov.<br>Not used in our analysis
nct_id	Identification number of the Study.<br>Not unique in this table as a study may have many publications associated with it
pmid	PubMed ID number.<br>In PubMed, this identifies a unique publication.<br>Not unique in this table as a PubMed publication may refer to several studies.
reference_type	One of 'background', derived' or 'result'.<br>From PubMed API:<br>ProtocolSection.ReferencesModule.ReferenceList.Reference.ReferenceType
citation	Citation list as presented in PubMed.<br>From PubMed API.<br>ProtocolSection.ReferencesModule.ReferenceList.Reference.ReferenceCitation<br>Not used in our analysis.

The combination of *nct_id* and *pmid* is unique in the table, except for cases where *pmid* is null.

[13] See: https://aact.ctti-clinicaltrials.org/data_dictionary#tableDictionary, Name="study_references".

SQL to extract nct_id and pm_id links:

The SQL below attempts to extract all 3378 *nct_id*s from the AACT reporting database in our base set.

```
select nct_id, pmid::integer as pm_id, reference_type
  from study_references
 where reference_type in ('result', 'derived')
   and pmId is not null
   and nct_id in (
    'NCT01246882', 'NCT01177774', 'NCT01119703', 'NCT01066208', 'NCT01023243',
    -- 3370 nct_ids removed for documentation.
    -- See the original SQL file for full list.
    'NCT00904007'
   )
```

Note:

- The full SQL for this is provided in
- many *nct_ids* did not appear in the STUDY_REFERENCES table.
- duplicate *nct_ids* and duplicate *pm_ids* are valid in the set, but not duplicate pairs.

Our analysis applied the following filters:

- *nct_id* contained in the *initial_study_set*.
- *pmid* is not null
- *reference_type* = 'result' or 'derived'

Appendix 2. VBA - Calculating *SearchKeysFound*

A Microsoft Excel User Defined Function was written to determine if the string pAbstract contains any of the values listed in the KeyPhrases collection.

(The full code for this module is available in the file vba_Module1.bas)

Note: when calling this function from the spreadsheet, we concatenated the *title* and *abstract* fields as the input variable.

```
Function searchForKeyPhrases(pAbstract As String) As Variant
    ' Main search function to find search terms associated with AI/ML
```

```
' Don't calculate for empty text
If Len(pAbstract) < 2 Then
    searchForKeyPhrases = ""
    Exit Function
End If

Dim KeyPhrases As New Collection

' Add key phrases to the Collection
With KeyPhrases
    .Add "ai-based"
    .Add "artificial intelligence"
    .Add "augmented intelligence"
    .Add "deep learning"
    .Add "convolutional neural network*"
    .Add "deep neural network*"
    .Add "artificial neural network*"
    .Add "recurrent neural network*"
    .Add "generative adversarial network*"
    .Add "deep reinforcement learn*"
    .Add "machine learning"
    .Add "bayesian network* "
    .Add "classification tree*"
    .Add "gradient boosting"
    .Add "XGBoost"
    .Add "multilayer perceptron*"
    .Add "naïve bayes"
    .Add "naive bayes"
    .Add "k nearest neighbor*"
    .Add "random forest"
    .Add "regression tree"
    .Add "vector machine"
    .Add "natural language?processing*"
    .Add "reinforcement learn*"
    .Add "supervised learn*"
    .Add "swarm intelligence"
    .Add "unsupervised learn*"
End With

Dim vMatchList As String
Dim count As Integer
count = 0
```

```
    vMatchList = ""

    Dim pattern As String
    Dim regEx As Object
    Set regEx = CreateObject("VBScript.RegExp")
    regEx.Global = True
    regEx.IgnoreCase = True


    ' Loop through all the KeyPhrases in the collection
    Dim phrase As Variant
    For Each phrase In KeyPhrases
        ' Create a regex pattern, replacing spaces with a regex pattern for
whitespace/punctuation
        pattern = Replace(phrase, " ", "[\s\/\-_&]+")
        regEx.pattern = pattern

        ' Search for the pattern in the abstract
        If regEx.Test(pAbstract) Then
            vMatchList = vMatchList & phrase & "|"
            count = count + 1
        End If
    Next phrase

    ' Handle case of no matches
    If count = 0 Then
        vMatchList = "No keys found!"
    ElseIf count > 0 Then
        ' Remove the last pipe character
        vMatchList = Left(vMatchList, Len(vMatchList) - 1)
    End If

    searchForKeyPhrases = vMatchList

End Function ' searchForKeyPhrases
```

Appendix 3. VBA for PubMed and Scopus APIs

Visual Basic for Applications programs were written to perform the data extracts from PubMed and Scopus. Data tables were contained in Microsoft Excel worksheets, and extracted data was written into the output tables in other worksheets.

VBA “Step 3 PubMedApi.bas” module

This module contains two main programs.
(The full code for this module is available in the file “Step 3 PubMedApi.bas”)

get_PM_batch_from_nct_list()

Iterates through the Excel table which contains data from the *initial_nct_id_set*, and presents each *nct_id* to the PubMed “esearch” API. The API returns an XML with a list of any *pm_ids* that were found for the target *nct_id*. If data is found, it is written to another list within the Excel workbook.

API Calls:

https://eutils.ncbi.nlm.nih.gov/entrez/eutils/esearch.fcgi?db=pubmed&term=""" & pSearchValue & """"

main2_Date_and_Abstracts()

Once the full list of *pm_ids* has been compiled, this program iterates through the list and presents each *pm_id* to the PubMed “esummary” API. It then extracts the Title and Publication Date from the XML. A subsequent call to the “efetch” API extracts the publication’s abstract.

API Calls:

https://eutils.ncbi.nlm.nih.gov/entrez/eutils/efetch.fcgi?db=pubmed&rettype=abstract&retmode=text&id=" & pPM_id
"https://eutils.ncbi.nlm.nih.gov/entrez/eutils/esummary.fcgi?db=pubmed&version=2.0&id=" & pPM_id

VBA “Step 4 ScopusApi.bas” module

(The full code for this module is available in the file “Step 4 ScopusApi.bas”)

get_Scopus_batch_from_nct_list()

iterates through the Excel table which contains data from the *initial_nct_id_set*, and presents each *nct_id* to the Scopus API. The API returns an XML with a list of any *pm_ids* that were found for the target *nct_id*.
In many cases, Scopus found the *nct*_id, but no *pm_ids* were found in the XML. In such cases, we extracted the publications doi and presented this to the PubMed “esearch” API.
If data is found, it is written to another list within the Excel workbook.

API Calls:

"https://api.elsevier.com/content/search/scopus?query=title-abs(" & pSearchValue & ")&apiKey=" & g_Scopus_ApiKey

https://eutils.ncbi.nlm.nih.gov/entrez/eutils/esearch.fcgi?db=pubmed&term=""" & pDOI & """"

vba_tools.bas module

This module contains various tools used in the PubMed and Scopus modules.

Particularly tools for manipulating XML files, and working with API calls.

(The full code for this module is available in the file vba_Tools.bas)

Appendix 4. Python Program to retrieve PubMed MeSH and Keyword attributes.

(The full code for this module is available in the file "*fetch_pubmed_xml.py*")

A python program was written to iterate through the total list of PM_IDs to obtain the MeSH Heading and Keyword nodes from the publications XML record.

The program called the PubMed eFetch API, using the XML options, rather than the Text options used in the VBA:

https://eutils.ncbi.nlm.nih.gov/entrez/eutils/efetch.fcgi?db=pubmed&id={pmid}&retmode=xml

```
# MDM 2024-12-22 - Pulls eFetch XML from PubMed
# MDM 2024-12-25 - Extracts MeSH, Keywords, Title, Abstract
#                , Authors (combined names), and saves results in Excel.
#                  Includes Excel write checks, overwrites existing data
#                , saves XML in pretty format, and rate-limits requests.

import requests
import os
import xml.etree.ElementTree as ET
from xml.dom.minidom import parseString
from openpyxl import Workbook
import sys
from colorama import init, Fore
import platform
import time
from datetime import datetime
import xml.etree.ElementTree as ET
```

```
# --------------------------------
# Configuration
# --------------------------------
# List of PMIDs
pmid_list = [
     '152'
   , '570666'
   , '843779'
   , '1058213'
#    …    rows removed, see attached file for full program.
   , '38296269'
   , '38299639'
   , '38326352'
]

# Directory for saving XML files
output_dir = r"D:\Work\!!2024-12\PubMedXml"
xml_dir    = r"D:\Work\!!2024-12\PubMedXml\xmlFiles"

# Excel output file
excel_path = os.path.join(output_dir, "PubMed_eFetch_Results.xlsx")

# Rate limiter configuration
rate_limit_delay = 3  # Delay in seconds (after every batch)
rate_limit_batch = 40  # Process this many rows before pausing

# Initialize colorama for colored text
init(autoreset=True)
```

```
# -------------------------------
# Helper Functions
# -------------------------------

# Beep sound for errors
def beep():
    if platform.system() == "Windows":
        import winsound
        winsound.Beep(1000, 500)  # Frequency = 1000Hz, Duration = 500ms
    else:
        os.system('echo -e "\a"')  # Linux/Mac beep

# Check if Excel file is writable
def check_excel_writable(filepath):
    try:
        if os.path.exists(filepath):
            with open(filepath, "a"):  # Try opening the file in append mode
                pass
        return True
    except PermissionError:
        print(Fore.RED + f"Excel file '{filepath}' is locked or open. Close it and
try again.")
        beep()
        sys.exit(1)

# Extract Title
def extract_title(root):
    title = root.find(".//ArticleTitle")
    return title.text if title is not None else "None"

# Extract Abstract
def extract_abstract(root):
    abstract_text = []
    for abstract in root.findall(".//AbstractText"):
        label = abstract.get('Label', '')
        text = abstract.text or ''
        if label:
            abstract_text.append(f"{label}: {text}")
        else:
            abstract_text.append(text)
    return " ".join(abstract_text) if abstract_text else "None"

# Extract MeSH headings
def extract_mesh_headings(root):
```

```
    mesh_headings = []
    for mesh in root.findall(".//MeshHeading/DescriptorName"):
        mesh_headings.append(mesh.text)
    return "~".join(mesh_headings)

# Extract Keywords
def extract_keywords(root):
    keywords = []
    for kwd in root.findall(".//Keyword"):
        keywords.append(kwd.text)
    return "~".join(keywords)

# Extract Author Names (combined as LastName, Initials)
def extract_authors(root):
    authors = root.findall(".//Author")

    # Helper to format name as "LastName, Initials"
    def format_name(author):
        if author is not None:
            last_name = author.find("LastName").text if author.find("LastName") is
not None else "None"
            fore_name = author.find("ForeName").text if author.find("ForeName") is
not None else ""
            initials = "".join([x[0] for x in fore_name.split()])  # Extract
initials
            return f"{last_name}, {initials}" if initials else last_name
        return "None"

    # First and Last Authors
    first_author = format_name(authors[0]) if authors else "None"
    last_author = format_name(authors[-1]) if authors else "None"

    return first_author, last_author

def get_earliest_date(root):
    # Extracts the earliest publication date from an eFetch XML response.
    # Searches PubMedPubDate, EPubDate, PubDate, ReceivedDate, and AcceptedDate.
    # Returns 'Missing' if no valid dates are found.
    def parse_date(date_str):
        # Helper to parse dates safely, defaulting to 1st if parts are missing.
        try:
            parts = date_str.split("-")
            year = int(parts[0])
            month = int(parts[1]) if len(parts) > 1 else 1
            day = int(parts[2]) if len(parts) > 2 else 1
```

```
            return datetime(year, month, day)
        except Exception:
            return None

    # Collect all potential dates
    pub_dates = []

    # 1. PubMedPubDate nodes (Primary dates for PubMed indexing)
    for pub_date in root.findall(".//PubMedPubDate"):
        status = pub_date.get("PubStatus")
        if status in ["pubmed", "entrez"]:
            year = pub_date.find("Year").text if pub_date.find("Year") is not None
else "9999"
            month = pub_date.find("Month").text if pub_date.find("Month") is not
None else "01"
            day = pub_date.find("Day").text if pub_date.find("Day") is not None
else "01"
            pub_dates.append(parse_date(f"{year}-{month}-{day}"))

    # 2. EPubDate (Electronic publication date)
    epub_date_node = root.find(".//EPubDate")
    if epub_date_node is not None and epub_date_node.text:
        epub_date = parse_date(epub_date_node.text.replace("/", "-"))
        if epub_date:
            pub_dates.append(epub_date)

    # 3. PubDate (General publication date)
    pub_date_node = root.find(".//PubDate")
    if pub_date_node is not None and pub_date_node.text:
        pub_date = parse_date(pub_date_node.text.replace("/", "-"))
        if pub_date:
            pub_dates.append(pub_date)

    # 4. AcceptedDate (Editorial acceptance date)
    accepted_date_node = root.find(".//AcceptedDate")
    if accepted_date_node is not None and accepted_date_node.text:
        accepted_date = parse_date(accepted_date_node.text.replace("/", "-"))
        if accepted_date:
            pub_dates.append(accepted_date)

    # 5. ReceivedDate (Submission date)
    received_date_node = root.find(".//ReceivedDate")
    if received_date_node is not None and received_date_node.text:
        received_date = parse_date(received_date_node.text.replace("/", "-"))
        if received_date:
```

```
            pub_dates.append(received_date)

    # Return the earliest valid date or 'Missing'
    if pub_dates:
        earliest_date = min([date for date in pub_dates if date is not None])
        return earliest_date.strftime("%Y-%m-%d")
    else:
        return "Missing"

# --------------------------------
# Main Program
# --------------------------------

# Ensure output directory exists
os.makedirs(output_dir, exist_ok=True)

# Check Excel file writability
check_excel_writable(excel_path)

# Overwrite Excel file (restart data)
wb = Workbook()
ws = wb.active
ws.title = "PubMed Data"
# Write header row
ws.append(["PMID", "PubDate", "Title", "Abstract", "MeSH Headings", "Keywords",
           "First Author", "Last Author"])

# Loop through PMIDs
total_rows = len(pmid_list)
for index, pmid in enumerate(pmid_list, start=1):
    try:
        # Print progress
        percent_complete = (index / total_rows) * 100
        print(f"Row {index}/{total_rows} ({percent_complete:.2f}%): Processing PMID
{pmid}")

        # Fetch data from PubMed
        url =
f"https://eutils.ncbi.nlm.nih.gov/entrez/eutils/efetch.fcgi?db=pubmed&id={pmid}&ret
mode=xml"
        response = requests.get(url)

        if response.status_code == 200:
            # Pretty format XML and save
            file_path = os.path.join(xml_dir, f"{pmid}.xml")
```

```
            pretty_xml = parseString(response.text).toprettyxml()
            with open(file_path, "w", encoding="utf-8") as file:
                file.write(pretty_xml)

            # Parse XML
            root = ET.fromstring(response.content)

            # Extract fields
            vTitle = extract_title(root)
            vAbstract = extract_abstract(root)
            vMeSH_Heading_List = extract_mesh_headings(root) or "None"
            vKeywordList = extract_keywords(root) or "None"
            vFirstAuthor, vLastAuthor = extract_authors(root)

            vEarliestDate = get_earliest_date(root)

            # Write data to Excel
            ws.append([pmid, vEarliestDate, vTitle, vAbstract, vMeSH_Heading_List,
vKeywordList,
                       vFirstAuthor, vLastAuthor])

            # Rate limit every x rows
            if index % rate_limit_batch == 0:
                print(Fore.YELLOW + f"Pausing for {rate_limit_delay} seconds...")
                time.sleep(rate_limit_delay)

        else:
            raise Exception(f"HTTP Status {response.status_code}")

    except Exception as e:
        beep()
        print(Fore.RED + f"Error processing PMID {pmid}: {str(e)}")

# Save Excel filex
wb.save(excel_path)

print(Fore.GREEN + f"Excel file saved: {excel_path}")
print("Process complete!")
```
